# Evolved Features of Artistic Motivation: Analyzing a Brazilian Database Spanning Three Decades

**DOI:** 10.3389/fpsyg.2021.769915

**Published:** 2021-12-21

**Authors:** Marco Antonio Correa Varella

**Affiliations:** Department of Experimental Psychology, Institute of Psychology, University of São Paulo, São Paulo, Brazil

**Keywords:** arts, aesthetic behavior, motivation, career-choice, evolutionary psychology, domain-specificity, creativity, creative professions

## Abstract

Darwin explored the evolutionary processes underlying artistic propensities in humans. He stressed the universality of the human mind by pointing to the shared pleasure which all populations take in dancing, engaging in music, acting, painting, tattooing, and self-decorating. Artistic motivation drives/reinforces individuals to engage in aesthetically oriented activities. As curiosity/play, artistic behavior is hypothesized as a functionally autonomous activity motivated intrinsically through an evolved, specific, and stable aesthetic motivational system. The author tested whether artistic motivation is rather intrinsically sourced, domain-specific, and temporally stable using a large decades-long real-life public Brazilian database of university applications. In Study I, the author analyzed reasons for career-choice responded to by 403,832 late-adolescent applicants (48.84% women), between 1987 and 1998. In Study II, the author analyzed another career-choice reason question responded to by 1,703,916 late-adolescent applicants (51.02% women), between 1987 and 2020. Music, Dance, Scenic Arts, Visual Arts, and Literary Studies, in combination, presented a higher percentage of individuals reporting intrinsic factors (e.g., personal taste/aptitude/fulfillment) and the lower proportion reporting extrinsic motives (e.g., the influence of media/teacher/family, salary, social contribution/prestige) than other career groups. If artistic motivation were a recent by-product of general curiosity or status-seeking, artistic and non-artistic careers would not differ. Overall, intrinsic motives were 2.60–6.35 times higher than extrinsic factors; among artistic applicants’ were 10.81–28.38 times higher, suggesting domain-specificity. Intrinsic motivation did not differ among artistic careers and remained stable throughout the periods. Converging results corroborated a specific, stable, and intrinsically sourced artistic motivation consistent with its possible evolutionary origins.

## Introduction

In Darwin’s currently sesquicentennial book “The Descent of Man, and Selection in Relation to Sex” (1871), he not only expands his theorization about human origins and common descent, but also addresses the evolution of many psychobehavioral tendencies of our species (Campbell, 1972; Richerson et al., 2021). The evolution of our artistic propensities was an important part of the book (Menninghaus, 2019). He stressed the artistic universality by saying that the close similarity between all human populations in “tastes, dispositions and habits” is revealed by “the pleasure which they all take in dancing, rude music, acting, painting, tattooing, and otherwise decorating themselves” (p. 232). Darwin considered the human aesthetic and artistic tendencies both influenced by cultural and evolutionary factors, so he has opened fruitful lines of research that have since flourished.

Over the last decades, a growing body of literature considers artistic tendencies as part of human natural endowment, focusing on aesthetics (Voland and Grammer, 2003; Chatterjee, 2013), music, and dance (Wallin et al., 2000; Mithen, 2005; Ball, 2010; Bannan, 2012; Honing, 2018), literature and poetry (Carroll, 1995; Gottschall and Wilson, 2005; Boyd, 2009; Gottschall, 2012), visual arts (Coe, 2003), and arts in general (Dissanayake, 1988, 1992, 2000; Eibl-Eibesfeldt, 1989; Martindale et al., 2007; Dutton, 2009; Davies, 2012; Sütterlin et al., 2014; Hogh-Olesen, 2018; Menninghaus, 2019; Richards, 2019). However, there are also non-adaptive hypotheses, in which artistic activities would be a pleasurable technology (i.e., an unspecialized by-product of pre-existing capacities recently co-opted) (Pinker, 2004; Souza, 2004; Panksepp, 2009; Patel, 2010; Verpooten and Nelissen, 2012; Hodgson and Verpooten, 2015). To move beyond eventual “just-so stories” (Varella et al., 2013) and anecdotal evidence (Varella et al., 2017) on both sides, more high-quality empirical studies are still needed to tell apart adaptive and non-adaptive hypotheses about artistic propensities (cf. Andrews et al., 2002; Varella et al., 2012).

Many definitions of artistic-like activities stress manipulations performed to capture the attention, emotion, and imagination of others (cf. Varella et al., 2011, 2017). Varella et al. (2011) put together four independent lists of specific manipulations that artists employ to aesthetically enhance objects and behaviors to super-stimulate perceivers’ appreciation. In effect, the interpersonal dynamics of artistic activities are akin to the multimodal signaling and communicative process (Varella et al., 2011; Valentova et al., 2017, 2019; De Tiège et al., 2021). The stimulating artistic activities are a result of human “artisticality”; a term that generically encompasses the propensities to ontogenetically develop psychological faculties that underlie a whole array of multimodal and extraordinary aesthetically enhancing activities, including behaviors, its products, and appreciations across cultures, historical periods, and species (Varella et al., 2017; Varella, 2018).

As for musicality (Bispham, 2009), artisticality has at least three main psychological components: capacities for (re)creative production (executive), aesthetic appreciation (perceptive), and artistic motivations (drive) (Varella et al., 2011, 2017; Watanabe, 2013). Artistic motivations are related to “the pleasures that we all take in” (p. 232) engaging in aesthetic/artistic activities, as Darwin (1871) has stressed. Artistic motivations drive and reinforce individuals to start and maintain engagement in artistic activities, both production and appreciation. Artistic motivation rewards and impels individuals to use their level of artisticality to actively or passively engage in aesthetically oriented activities with some level of commitment. Artistic motivation concerns both the drive to directly reach the final goal of producing and appreciating arts, and the drive to reach non-artistic commitments and sub-goals (e.g., face the traffic jam to visit the art museum, or to pass exams for an artistic career) which lead to the final goal of appreciating and producing arts.

Motivation can be broadly described as a modulating and coordinating influence on the direction, vigor, and composition of behavior that arises from a wide variety of internal, environmental, and social sources, and is manifested at many levels of the behavioral and neural organization (Shizgal, 2001). Traditionally, motivating factors are broadly divided into internal and external. Activities that provide their own inherent reward are considered intrinsically motivated (Deci and Ryan, 2000; Ryan and Deci, 2017). Whereas, extrinsic motivation refers to individuals’ engagement in an activity to obtain some instrumentally distinct consequence, such as money or status (Deci and Ryan, 2000; Ryan and Deci, 2017).

The existence of specific feelings, desires, pleasures, or intrinsic motivation for a given activity possibly signals ancestral biological advantages and can be seen as an indicator of its evolved nature (Thornhill, 2003; Huron, 2005). To be converted into behavioral action, the outputs of cognitive mechanisms require motivational processes (Berridge, 2012; Anselme, 2016), which coevolve with the respective cognition (Ermer et al., 2008). The functional/adaptive significance of a given set of behaviors is dependent on the underlying cognitive capabilities and on the factors that motivate/sustain performance in appropriate contexts (Bispham, 2009). Thus, the behavioral expression of appreciative and executive artistic capacities needs to have a coevolved correspondent underlying motivational system. Uncovering the motivational basis of artistic propensities is a fundamental aspect of evolutionary theorization (cf. Merker et al., 2015).

Similar to curiosity and play, artistic behavior can be hypothesized as a functionally autonomous activity motivated rather intrinsically through an evolved and specific aesthetic motivational system. For instance, Morris (1962) concludes that the shared picture-making motivation in apes and humans has an element of self-rewarding activation. Csikszentmihalyi (1996) conceptualizes creativity as an autotelic activity that is enjoyable in itself. Watanabe (2013) concludes that the self-reinforcing power of the art-like behavior and the artistic product for the artist and conspecifics are mostly unique to humans. Darwin (1871) and Dutton (2009) considered the direct and universal artistic pleasure, and (Dutton, 2009) concluded that it is one of the main evolved features of human artistic instinct. Hogh-Olesen (2018) refers to the universal necessity to embellish the human body and surroundings and the desire to fill time and space with song, music, dance, and stories as the aesthetic impulse, a primary impulse inherent to human nature. These do not exclude the possibility that some external factors, such as social belonging or mate attraction, coul has stressed. Artistic motivations d also be important, and possibly even evolved, extrinsic motivations activating artistic tendencies (cf. Winegard et al., 2018).

Different lines of evidence seem to suggest the existence of an intrinsic motivational drive underlying artistic manifestation. Some evidence shows that a deep motivation toward artistic self-expression is uninhibited when the communicative channel of language fails following neurological damage (Zaidel, 2014). In the majority of these neurological cases, patients increase the production of art, occasionally prolifically, emerging even if never previously expressed (i.e., *de novo* artist) (cf. Midorikawa and Kawamura, 2015), despite the damage’s laterality or localization (Zaidel, 2014). The emergence of *de novo* artists after brain damage is not short-lived and the engagement in artistic expression tends to be compulsive and highly sustained (Abraham, 2019).

Another strategy to assess the importance of intrinsic motivation is to administer an external reward and evaluate whether it has negative effects on the behavior. Morris (1962) described when a chimpanzee once received food reward as drawing encouragement. However, the drawing aesthetic value was impaired; the ape took less interest in the drawing lines; any scribble would do and then it would immediately hold out its hand for the extrinsic reward. Similarly, intrinsically motivated paper collages done by 7–11 years old girls were evaluated as higher on creative expression than the extrinsically motivated group, which, in turn, was higher on the technical level (Amabile, 1982). Moreover, collages made by preschool children with a free choice of materials were judged by artists as more creative than those made by children for whom the experimenter chose the materials (Amabile and Gitomer, 1984). Further, non-rewarded artistic activity (collage-making, storytelling) is evaluated as more creative than the rewarded activity among both children and adults (Amabile et al., 1986).

Intrinsic motivation is also frequently assessed in terms of freely pursued activities. According to Cushman and Laidler (1990), leisure activity is pleasurable and intrinsically motivated, being an end in itself and valuable for its own sake. Aesthetics and artistic activities are among the most frequently spontaneously pursued free-time activities and hobbies (Kleiber et al., 1986; Chalip et al., 1996; Walker and Scott-Melnyk, 2001; Chamorro-Premuzic and Furnham, 2004; Furnham and Chamorro-Premuzic, 2004; McManus and Furnham, 2006; Mosing et al., 2015).

Intrinsic and extrinsic motivations can be explored by analyzing the explicit reasons for individuals’ engagement with activities. Frequent participants of arts and cultural activities declare they want to support important community organizations and events through their participation, learn more about other cultures, and experience high-quality art (Walker and Scott-Melnyk, 2001). McCarthy and Jinnett (2001) found that personal interest in the artistic material itself, the desire to express themselves artistically, opportunity for social interaction, interest in learning more about the arts, education, enrichment, and accompanying a friend or a family member are among the reasons for taking part in artistic organizations. Swanson et al. (2008) found that recreation, aesthetics, social interaction, escape, education, and self-esteem were the reasons for attending performances of theater, comedy, and vocal popular music. Chong (2010) found that the reasons for enjoying singing were self-expression, aesthetic experience, interpersonal relationships, stress reduction/mood change, spirituality, empowerment/identity, and self-actualization. Despite not explicitly distinguishing between intrinsic and extrinsic factors, notably, some intrinsic factors are among the top-ranked reasons followed by some extrinsic factors related to social interaction. These findings strengthen the case for an evolved rather intrinsic artistic motivation.

Further, modularity, domain-specificity, or functional autonomy/specialization have been considered a hallmark of mental adaptation because different information-processing problems usually require different procedures for their successful solution (Cosmides and Tooby, 1994; Andrews et al., 2002; Barrett, 2008; Tooby and Cosmides, 2015). Thus, specialization was selected because it pays off in terms of solving problems faster and more efficiently (Cosmides and Tooby, 1994; Tooby and Cosmides, 2015). Watanabe (2013) stresses the functional autonomy of art-like behavior among animals, particularly in humans. Although no study has focused on how specific is artistic motivation, the cases of *de novo* artists following brain damage (e.g., Abraham, 2019), the cases of exceptional and unexpected artistic talent in young autistic individuals dissociated from their learning difficulties (e.g., Gordon, 2005; Zaidel, 2014; Paola et al., 2020), the domain-specificity of musical and aesthetic creativity (Feist, 2004), and the spontaneous choices of artistic leisure activities (e.g., McManus and Furnham, 2006) together indicate some level of specificity in artistic motivation. Thus, it is plausible that artistic motivations would present signs of domain-specificity.

Furthermore, the comparative evidence of homology indicates a deep-seated stable motivational tendency for aesthetic display. Great apes and humans share an element of self-rewarding activation during picture-making (Morris, 1962). Captive male orangutans spontaneously perform premeditated long nocturnal calls (Samson et al., 2014). Chimpanzees exhibit a spontaneous tendency toward entrainment and rhythmic synchronization (Hattori et al., 2013; Large and Gray, 2015), and in their natural environment, chimpanzees exhibit episodes of rain-dancing, pant-hoot chorusing, or “carnival displays” (Dufour et al., 2015). No study has investigated the temporal stability or long-term trends of artistic motivation; however, artistic interest is stable over individual ontogeny (e.g., Waller et al., 1995), early paleo art is antique and its occurence is somewhat constant since Middle Paleolithic (Henshilwood et al., 2018), and artistic manifestations have universal aspects, which indicates a stable motivational core for this tendency across-cultures (Brown, 1991; Dissanayake, 2008). Hence, it is also probable that artistic motivation is to a certain degree, temporally stable across generations, possibly detectable even in small time scales of a few decades.

Although corroborating, the existing literature has many limitations that hinder wide-ranging conclusions about the psychological structure of artistic motivation, regarding its source, specificity, and stability. Previous studies focus on a limited range of artistic activities, have relatively small samples, and tend to be restricted to one or a few North American or West European locations. This is far from representative of the diversity of human species (Henrich et al., 2010), which requires a more inclusive science of the human mind across cultures (Barrett, 2020), focusing particularly on less-studied populations such as in Africa and Latin America (Rad et al., 2018). In order to overcome these limitations, I investigated reasons for artistic commitment using a decades-long real-life massive database from late adolescents applying to a public university in Brazil.

## The Present Study

I tested whether artistic motivation is rather intrinsic, domain-specific, and temporally stable using explicit reasons for choosing a career through a university degree. Individual variation in vocational interests is among the most stable of all psychological constructs (Low et al., 2005), and during young adulthood there is an increase in interests related to “people” which comprises artistic, social, and enterprising (Hoff et al., 2018). Career choice is associated with many factors including individual cognitive style (Varella et al., 2016). The choice of artistic careers is a real-life situation and a crucial intermediary step toward an increased commitment to and engagement with artistic activities.

There is a socio-demographic questionnaire required upon registering for the entrance exams which had two multiple-answer questions about motives/reasons for choosing the main career: Study I focuses on the short-lived question (1987–1998) and Study II addresses the longstanding question about reasons for career application (1987–2020). The comparison among different classes of reasons for applying to a university degree indicates whether artistic motivation is more intrinsically or extrinsically sourced. The comparison between artistic and non-artistic careers and within artistic careers allows measuring how specific is the artistic motivational profile. The correlation between the examination year and classes of reasons indicates possible long-term temporal trends or stability (cf. Varella et al., 2016).

A non-adaptive hypothesis would likely predict that the intrinsic motivational factors related to artistic activities would be a direct and undifferentiated by-product of general curiosity. As such, it would predict no difference from non-artistic careers since curiosity arguably contributes to the choice of all careers. Further, Pinker (2004) stated that art (other than narrative) could be a by-product of three distinct pre-existing mental adaptations: the aesthetic pleasure of experiencing adaptive objects and environments (perception); the ability to design artifacts to achieve desired ends (production); and the hunger for status (motivation). Thus, according to Pinker (2004), the artistic activity would not be an end in itself, but rather another means to obtain social status and prestige. If that is true, I would find more extrinsic factors such as “influence of the family,” “influence of the educational advisor,” “social prestige,” and “job market,” and “ample possibilities of salary” as the main reasons for seeking artistic careers. Moreover, since the general hunger for status can possibly influence the choice of any career, there would be no profile difference between artistic and non-artistic careers. Finally, because the popularity and prestige of careers can change over the years, the non-adaptive hypothesis would predict that artistic motivation to be as influenced by historical and societal changes as non-artistic careers exhibiting long-term trends and being temporally unstable within the three decades’ timeframe.

### Aims

I tested whether artistic motivation is rather intrinsic, domain-specific, and temporally stable, as predicted for an evolved trait. Based on the above-mentioned literature, artistic activity is hypothesized as a functionally autonomous activity motivated rather intrinsically through an evolved, specific, and temporally stable underlying aesthetic motivational system. However, if the main motivational factors are general curiosity and/or general status-seeking, it is hypothesized that artistic motivation would be balanced between intrinsic and extrinsic factors, there would be no differences between artistic and non-artistic careers, and the interest in artistic career would trend upwards or downwards along with non-artistic careers following recent socio-historical factors. The author examined reasons for applying to an artistic career using a decades-long real-life massive database from late adolescents applying to a prominent public university in Brazil.

## Study I

### Materials and Methods

The author accessed public data available online from the *Comissão Permanente para os Vestibulares* (COMVEST) website.^1^ COMVEST runs the entrance examinations to the University of Campinas (UNICAMP), one of the three main public São Paulo State universities (Brazil). UNICAMP is one of the most desired places to study and it involves real-life choices and implications, which gives an ecologically powerful measure of motivation and vocation. At the website (e.g., for 1998)^2^ the data were collected from “vestibular” (i.e., university entrance exam), from “all cities,” “all kinds of public” (including “open concurrence” and “quotas”), and from all individuals “applying” for the entrance exam, not only those who were later approved and actually signed up for the chosen career, because being approved requires more than only interest/motivation (e.g., preparation, competitiveness, good previous education).

The short-lived question about motives/reasons to apply for a university career lasts 16 years (1987–2003), but after 1999 all answer options indicating intrinsic motivations were removed, which made the test comparing intrinsic and extrinsic reasons unfeasible. Thus, the author used data only until 1998 (11 years’ period). During this period, applicants could take the examination in up to 18 different medium-sized to large Brazilian cities (viz. Bauru, Campinas, Jundiaí, Limeira, Piracicaba, Presidente Prudente, Ribeirão Preto, Santo André, Santos, São José do Rio Preto, São Jose dos Campos, São Paulo, and Sorocaba, all from São Paulo State, and 5 other state capitals’: Brasília/DF, Belo Horizonte/MG, Curitiba/PR, Rio de Janeiro/RJ, and Salvador/BA). This includes four out of the five Brazilian regions and six out of the 26 Brazilian states. Between 1987 and 1998, the phrasing of this specific question was: “What is the predominant motive for your choice of the course [career] for which you are applying as the 1st option?” In Brazil, the application involves two or three options of careers ranked according to the interest of the applicant. The initial answer options were: “blank” (*missing*), “I’ve always liked it,” “research done at school,” “conversations with colleagues,” “information obtained from the communication media,” “influence of the family,” “results of vocational testing,” “influence of the teacher,” “influence of the educational advisor,” “the only one that is accessible to me,” and “other.” In 1989, the answer option “research done at school” was substituted by “personal aptitude for the fundamental courses of the career.” In 1991 the option “other” was removed from the COMVEST questionnaire, which did not change till 1998.

Between 1987 and 1998, 403,832 late-adolescent applicants (77.11% between 17 and 20 years old, 9.02% between 21 and 23 years old, 3.53% between 24 and 29 years old; 51.16% men and 48.84% women) answered about the single predominant reason for the first choice of the career application. Among the answer options, “I’ve always liked it” and “personal aptitude” were categorized as intrinsic motives, while “research done at school,” “conversations with colleagues,” “information obtained from the communication media,” “influence of the family,” “results of vocational testing,” “influence of the teacher,” “influence of the educational advisor,” and “the only one that is accessible to me” were categorized as extrinsic motives, following conceptualizations of Deci and Ryan (2000) and Ryan and Deci (2017). Even if the options categorized as extrinsic include some traces of intrinsic factors, to avoid confirmation bias the author kept as “intrinsic factor” only those options which show an unambiguous intrinsic aspect. Because there is a portion of participants that chose “other” or left it “blank,” the percentage of intrinsic and extrinsic categories do not necessarily add up to 100%. Table 1 presents the averaged percentage of choices for each answer option for all participants throughout the period.

**TABLE 1:** Averaged percentage of choices for each answer option for all participants between 1987 and 1998 in the basic order of appearance.

Answer options	Mean percentage of participants (Standard Deviation)
“Blank”	2.42% (2.36)
“I’ve always liked it”	46.39% (6.78)
“Personal aptitude”	32.78% (2.66)
“Research done at school”	0.22% (0.51)
“Conversations with colleagues”	1.58% (0.40)
“Information obtained from the communication media”	5,73% (0.96)
“Influence of the family”	1.52% (0.13)
“Results of vocational testing”	1.32% (0.32)
“Influence of the teacher”	0.42% (0.08)
“Influence of the educational advisor”	0.25% (0.08)
“The only one that is accessible to me”	2.13% (0.56)
“Other”	5.24% (0.57)

Online data was manually collected as the given percentage of individuals marking each answer option, including the option “blank,” for each year and career, tabulated into Excel along with the total individuals answering the questions. Using percentages controls for natural variation in the actual number of applicants in each career and year. Given the restricted nature of available data (i.e., percentage of individuals answering each option of each question), I do not have data per individual or sex, but only per year and career.

I collected data from all artistic careers offered at UNICAMP until 1998 (viz. Music, Dance, Scenic Arts, and Artistic Education). The different career modalities of music (viz. instrument [erudite], composition, teaching, conducting, and popular) were averaged together to create the overall Music career. All artistic careers contemplate both the analytical domain of history/theory/criticism and the creative production/performance domain. For comparison, I also collected data from some non-humanities careers (Chemistry, Dentistry, Physical Education), from some humanities non-artistic careers (History, Philosophy, Pedagogy, Social Sciences), and from “All careers” combined, which is the only aggregate option available online (44 different careers in 1998; treating Music modalities as two careers since most others are either integral and nocturnal or teaching and bachelor). The other aggregated groups (non-humanities, humanities non-artistic, and artistic) were created by averaging the percentage of respondents who chose each answer option across careers. Table 2 displays the numbers of individuals per career group throughout the period. Most mentioned careers had already existed since 1987. Philosophy and Popular Music started in 1989. The non-artistic careers for comparison were chosen to approximately match artistic careers in terms of the time of existence, sex composition, application interest, and perceived societal recognition/application. The same website (e.g., for 1998, see text footnote 2) contains those parameters among the many questions of the socioeconomic questionnaire. The chosen courses include classes in the morning and the afternoon (i.e., full-time), but not during the evening. Integral courses require longer daily dedication, are normally the oldest and the most preferred ones, which ensures more representative and larger samples.

**TABLE 2:** Number of individuals per career group from whom answers were accessed in the short-lived (1987–1998) question about reasons to apply for a career.

Career group	All careers	Non-humanities	Humanities non-artistic	Artistic
Number of	Between	403,832	48,778	15,536	10,163
individuals	1987 and 1998				

### Analyses and Results

After checking for transcription errors and creating each pooled averaged group of careers, the author created the two classes of motives (intrinsic and extrinsic) by adding the percentage of each correspondent and respective answer option (i.e., “I’ve always liked it” and “personal aptitude” as intrinsic and the remaining as extrinsic, see Table 1). The percentages of “blank” and “other” were ignored for their indeterminacy regarding the class of motives. Thus, intrinsic and extrinsic motives are not mere opposite measures, they do not even correlate [*r*(12) = -0.336, *p* = 0.285]. The author further created a ratio between the classes of motives by dividing the averaged intrinsic percentages by the averaged extrinsic percentages (i.e., In/Ex). This ratio indicates how many times intrinsic motives are higher than extrinsic motives, it creates a composite measure for an overall motivational profile, it enables group comparisons of the motivational profile, and it enables an integrated discussion of both intrinsic and extrinsic motives. Although the ratio of intrinsic to extrinsic factors is correlated to both with intrinsic [*r*(12) = 0.627; *p* = 0.029], and with extrinsic motives [*r*(12) = -0.942; *p* < 0.001], the degree of relationship is only strong enough to constitute a necessary condition for multicollinearity in the case of extrinsic factors (Schroeder et al., 1990). Further, the data was transferred into SPSS 20 (IBM Corp., Armonk, NY, United States) for group comparisons using the multivariate General Linear Model (GLM). To overcome this one case of multicollinearity, within the GLM I followed the more conservative Pillai’s Trace test, which has the highest power, thus being robust and indicated for this situation (Sarma and Vardhan, 2019). Bonferroni *post-hoc* test was used for main effects and Partial Eta-squared (η*_*p*_*^2^) as effect size estimator. I also performed Pearson correlation analyses. Zero percentages were treated as missing values because the COMVEST site presents zero percentages whenever there was any number of participants below five.

The author performed a multivariate GLM with the two classes of motives (intrinsic × extrinsic) and the intrinsic to extrinsic ratio as dependent variables, the groups of careers as independent factors (total of careers in the period, non-humanities careers, humanities non-artistic careers, and artistic careers), and the year (12 consecutive application period) as a covariate. The model [Pillai’s Trace = 1.045, *F*(12, 129) = 7.658, *p* < 0.001, η*_*p*_*^2^ = 0.348] showed that the combined artistic professions presented significantly higher percentage of individuals reporting intrinsic motives [*F*(3, 43) = 11.36, *p* < 0.001, η*_*p*_*^2^ = 0.442], lower percentage of individuals choosing extrinsic motives [*F*(3, 43) = 54.55, *p* < 0.001, η_*p*_^2^ = 0.792], and higher intrinsic to extrinsic motives ratio [*F*(3, 43) = 48.79, *p* < 0.001, η*_*p*_*^2^ = 0.773] than all other career groups.

**FIGURE 1:**
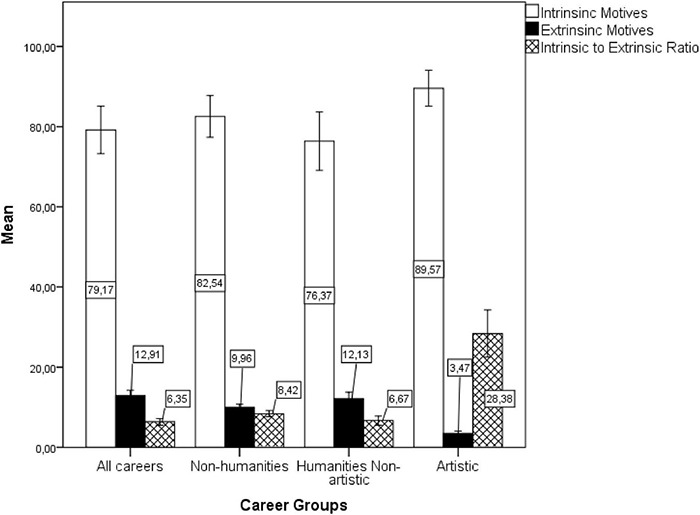
Averaged percentages of individuals indicating intrinsic or extrinsic motives of career choice by the cluster of careers in the short-lived question (1987–1998). Error bars are ± 2 Standard Errors (SEs).

The Bonferroni *post-hoc* test showed differences between Artistic careers and all other career groups in the intrinsic motives. Specifically, Artistic careers differed from All careers (*p* = 0.001), from non-humanities careers (*p* = 0.031) and from Humanities non-artistic careers (*p* < 0.001). Artistic careers further differed from all other careers in extrinsic motives (all *p*’s < 0.001), and in the intrinsic to the extrinsic ratio (all *p*’s < 0.001). Moreover, a lower percentage of individuals within non-humanities careers reported extrinsic motives for career choice than in the all non-artistic career group (*p* = 0.005). There was no other difference among the groups (see Figure 1). The intrinsic to extrinsic ratio showed that the total of applicants’ intrinsic motives (“I’ve always liked it” and “personal aptitude”) was 6.35 times higher than combined extrinsic factors (“research done at school,” “conversations with colleagues,” “information obtained from the communication media,” “influence of the family,” “results of vocational testing,” “influence of the teacher,” “influence of the educational advisor,” “the only one that is accessible to me”). The mean ratio between intrinsic and extrinsic motives was 8.42 for non-humanities and 6.67 for Humanities non-artistic. However, among the artistic career applicants’ intrinsic motives were 28.38 times higher than the extrinsic ones, suggesting a specific motivational profile.

In Figure 1, the cluster **“All careers”** includes up to 44 different careers; **“non-artistic”** includes Chemistry, Dentistry, Physical Education, History, Philosophy, Pedagogy, and Social Sciences; **“Non-humanities”** includes Chemistry, Dentistry, and Physical Education; **“Humanities Non-artistic”** includes History, Philosophy, Pedagogy, and Social Sciences; **“Artistic”** includes Music, Dance, Scenic Arts, and Artistic Education.

**FIGURE 2:**
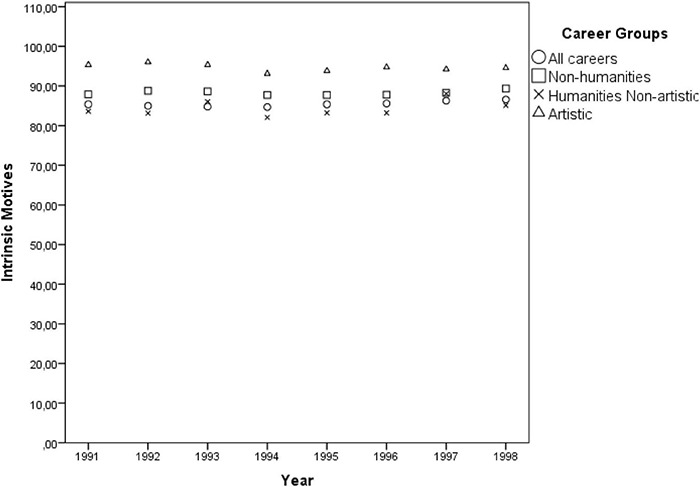
Dispersion of the averaged percentages of individuals indicating *intrinsic* motives of career choice by the cluster of careers throughout the years 1991 and 1998.

**FIGURE 3:**
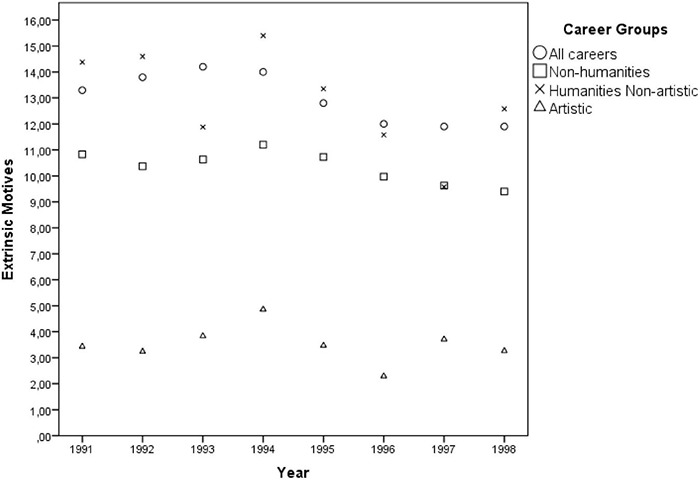
Dispersion of the averaged percentages of individuals indicating *extrinsic* motives of career choice by the cluster of careers throughout the years 1991 and 1998.

Although the multivariate GLM model [Pillai’s Trace = 0.671, *F*(3, 41) = 27.826, *p* < 0.001, η*_*p*_*^2^ = 0.671] has indicated a positive effect of the year only on intrinsic motives throughout the 12 consecutive application periods [*F*(1, 43) = 87.24, *p* < 0.001, η*_*p*_*^2^ = 0.670], the observed effect was an artifact of the official substitution of the answer option “research done at school” (extrinsic) by the option “personal aptitude” (intrinsic) in 1989 and the removal of the answer option “other” in 1991, which then probably induced more individuals to mark one of the popular intrinsic options. Thus, in order to account for these distortions, from the 12 consecutive application periods the author selected the 7 years after 1991 when the answer options remained unchanged. Pearson correlation showed that the years did not actually interact with intrinsic motivations [*r*(32) = 0.058, *p* = 0.752], neither with the extrinsic ones [*r*(32) = –0.151, *p* = 0.410], nor with the intrinsic to extrinsic ratio [*r*(32) = 0.071, *p* = 0.698], suggesting its overall temporal stability (see Figures 2, 3).

**FIGURE 4:**
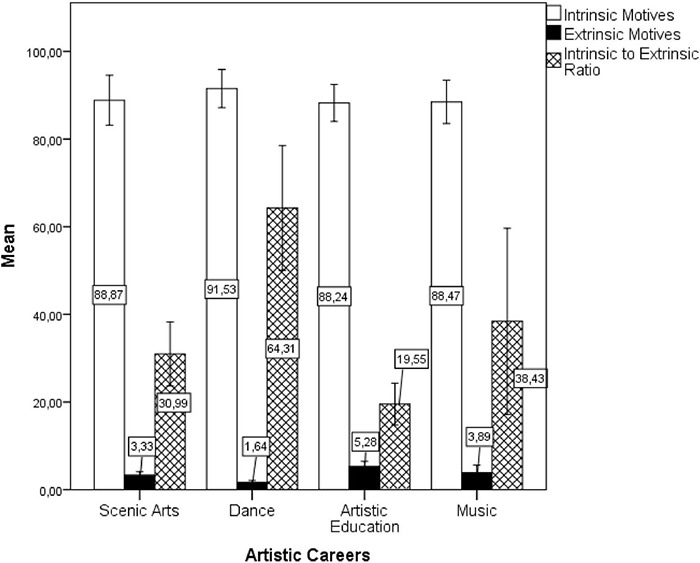
Averaged percentages of individuals indicating intrinsic or extrinsic motives of career choice by artistic careers in the short-lived question (1987–1998). Error bars are ± 2 SEs.

The author further compared the classes of motives among the artistic careers to test whether there is uniformity of pattern within the artistic career group and performed a multivariate GLM with the artistic careers as independent factors (Music, Dance, Scenic Arts, Artistic Education), the two classes of motives (intrinsic × extrinsic) and the intrinsic to extrinsic ratio as dependent variables, and the year (12 consecutive application periods) as a covariate. The model [Pillai’s Trace = 0.474, *F*(9, 123) = 2.562 *p* = 0.010, η*_*p*_*^2^ = 0.158] showed no difference among the artistic courses in the intrinsic motives [*F*(3, 41) = 1.22, *p* = 0.313, η*_*p*_*^2^ = 0.082], but showed differences in extrinsic motives [*F*(3, 41) = 6.06, *p* = 0.002, η*_*p*_*^2^ = 0.307] and in the intrinsic to extrinsic ratio [*F*(3, 41) = 7.22, *p* = 0.001, η*_*p*_*^2^ = 0.346]. The *post-hoc* with Bonferroni correction showed that a lower percentage of individuals reported extrinsic motives for Dance than for Artistic Education (*p* < 0.001). Moreover, Dance had higher intrinsic to extrinsic motives ratio than Scenic Art (*p* = 0.010) and Artistic Education (*p* < 0.001) (see Figure 4).

Although the multivariate GLM model has indicated a positive effect of the year on intrinsic motives throughout the 12 consecutive application periods [Pillai’s Trace = 0.607, *F*(1, 41) = 20.117, *p* < 0.001, η*_*p*_*^2^ = 0.607], again, there was no temporal effect after restricting analyses to the period when the answer options remained unchanged (between 1991 and 1998). The Pearson correlation between the 7 years after 1991 and the percentage of individuals reporting each class of motives or the intrinsic to extrinsic ratio showed no temporal changes to intrinsic motives [*r*(32) = -0.136, *p* = 0.459], to the extrinsic ones [*r*(30) = -0.058 *p* = 0.762], or to the intrinsic to extrinsic ratio [*r*(32) = 0.049, *p* = 0.792].

## Study II

### Materials and Methods

Similar to Study I, I assessed online the public data available at the COMVEST website. The collected data were the given percentage of individuals marking each answer option, including the option “blank,” for each year and career, and for “vestibular” (i.e., university entrance exams), “all cities,” “all kinds of public” (including “open concurrence” and “quotas”), and from “all applicants.” The applicants could take the examination in 35 different Brazilian cities until 2020, 30 from the São Paulo State, and capitals of 5 other Brazilian states (viz. MG, DF, PR, CE, and BA).

The long-lasting question about reasons to apply for a career between 1987 and 1998 was phrased as follows: “You chose the career or course for which you are applying as the 1st option based on:,” and the initial answer options were “blank” (*missing*), “job market,” “social prestige of the profession,” “adequacy for personal aptitude,” “low concurrence of the positions,” “ample possibilities of salaries,” “other motive.” Since 1989, the answer options “possibility of social contribution,” and “possibility of personal fulfillment” were added. Since 1999, the phrasing of the question turned to “What is the predominant motive for your choice of the course (career) for which you are applying as the 1st option?” and remained the same until nowadays, and the answer options remained the same. The only exception was the option “ample possibilities of salaries” that was permanently removed in 1999, possibly because of the lower adherence of respondents (see Table 3). The order of the answer options changed only once in 1999 and remained the same since then. Until 1999, “job market” appeared in second place (the current place of “personal aptitude”), and “personal aptitude” appeared in fourth place (the current place of “job market”). Although the exact specific phrasing of the question changed once, the main theme of the question and the majority of answer options remained basically the same throughout the 33 years.

**TABLE 3:** Descriptive statistics for the percentage of choice for each answer option for all participants between 1987 and 2020.

Answer option	Mean percentage of choice (Standard Deviation)
“Blank”	3.39% (7.75)
“Personal aptitude”	38.54% (10.10)
“Social prestige	2.00% (0.64)
“Job market”	8.43% (1.94)
“Social contribution”	14.20% (5.36)
“Lower concurrence”	1.10% (0.30)
“Ample possibilities of salary”	0.70% (0.57)
“Personal fulfillment”	26.20% (7.95)
“Other”	5.50% (3.44)

*This is the order in which the answer option appears between 1999 and 2020.*

Between 1987 and 2020, 1,703,916 late-adolescent applicants (84.52% between 17 and 20 years old, 9.14% between 21 and 23 years old, 4% between 24 and 29 years old; 48.98% men and 51.02% women) answered about the predominant reason for the first choice of the career application. Table 4 shows percentages of applicants per career group. Among the answer options, “personal aptitude” and “personal fulfillment” were categorized as intrinsic motives, while “social prestige,” “social contribution,” “lower concurrence,” “job market,” and “ample possibilities of salary” were categorized as extrinsic motives, following conceptualizations of Deci and Ryan (2000) and Ryan and Deci (2017). Again, the percentages of “blank” and “other” were ignored for their indeterminacy regarding the class of motives, thus although negatively correlated, [*r*(136) = –0.767; *p* < 0.001], intrinsic and extrinsic motives are not the mere opposite. Although the ratio of intrinsic to extrinsic factors is correlated to both with intrinsic, [*r*(136) = 0.661; *p* < 0.001], and with extrinsic motives [*r*(136) = –0.798; *p* < 0.001], the degree of relationship is not strong enough to constitute a necessary condition for multicollinearity (Schroeder et al., 1990). In 2020, the examinations occurred until the end of January, hence it was before the pandemic alert in Brazil which occurred in March. Table 3 displays averaged percentage of choice for each answer option for all participants throughout the period.

**TABLE 4:** The number of individuals per career group from which answers were accessed in long-standing (1987–2020) question about reasons to apply for a career.

Career group	All careers	Non-humanities	Humanities non-artistic	Artistic
Number of	Between	1,703,916	112,305	59,039	49,215
individuals	1987 and 2020				

I collected data from all artistic careers offered at UNICAMP so far (viz. Music, Dance, Scenic Arts, Artistic Education/Visual Arts, and Literary Studies). Artistic Education became Visual Arts in 2007, and Literary Studies was created in 2006. In the same way as the other artistic careers, Literary Studies also contemplates both the analytical domain of history/theory/criticism and the creative production/performance domain. After 1999, beyond the standard disciplines that were tested before the entrance, those applying for Music, Dance, Scenic Arts, and Artistic Education/Visual Arts had also required for each career a tailored theoretical and practical test of specific abilities. For comparison, I also collected data from the same comparison careers as in Study I (non-humanities careers: Chemistry, Dentistry, Physical Education; humanities non-artistic careers: History, Philosophy, Pedagogy, Social Sciences, and All careers combined—up to 62 in 2020). The pooled groups (non-humanities, humanities non-artistic, artistic, and overall Music) were created by averaging the percentage of respondents that had chosen each answer option across careers. Consistent with Study I, I also collected data only for full-time careers (i.e., including classes in the morning and the afternoon). Table 4 presents the number of individuals per career group throughout the period.

### Analyses and Results

Data treatment and statistical analyses were similar to Study I. The author checked for transcriptions errors, created each aggregate averaged group of careers, created the two classes of motives (intrinsic and extrinsic) by adding the percentage of each correspondent and respective answer option (i.e., “personal aptitude” and “personal fulfillment” as intrinsic and the remaining as extrinsic, see Table 3). The percentages of “blank” and “other” were ignored for their indeterminacy regarding the class of motives. The author further created the intrinsic to the extrinsic ratio which indicates how many times intrinsic motives are higher than extrinsic motives, creates a composite measure for an overall motivational profile, enables groups comparison of the motivational profile, and enables an integrated discussion of both intrinsic and extrinsic motives. Then, the data was transferred into SPSS 20 (IBM Corp., Armonk, NY, United States) for group comparisons using the multivariate GLM. To overcome another case of multicollinearity, within GLM I kept relying on the more conservative Pillai’s Trace test, which has the highest power (Sarma and Vardhan, 2019). Bonferroni *post hoc* test was used for testing the main effects, effect size estimator was Partial Eta-squared (η*_*p*_*^2^). The author ran Pearson correlation analyses. Zero percentages were considered missing values.

**FIGURE 5:**
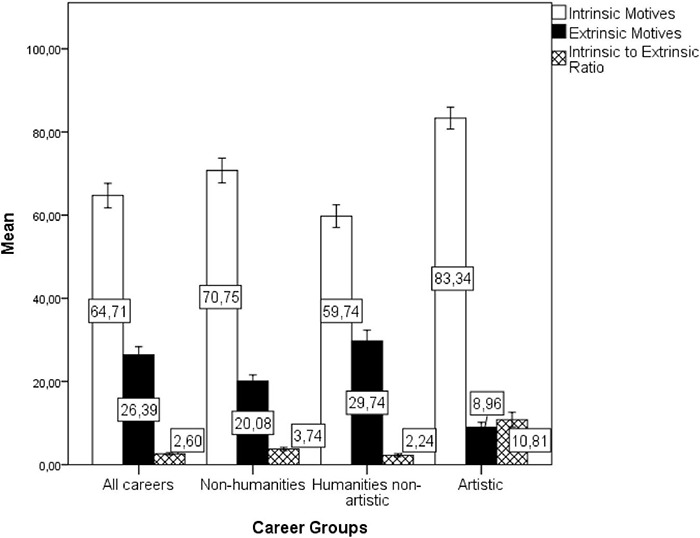
Averaged percentages of individuals indicating intrinsic or extrinsic motives of career choice by the cluster of careers in the long-standing question (1987–2020). Error bars are ± 2 SEs.

The author performed a multivariate GLM with the two classes of motives (intrinsic × extrinsic) and the intrinsic to extrinsic ratio as dependent variables, the groups of careers as independent factors (total of careers in the period, non-humanities careers, humanities non-artistic careers, and artistic careers), and the year (33 years’ period) as a covariate. The model showed that the career categories differ in intrinsic, extrinsic, and in the ratio of intrinsic to extrinsic factors [Pillai’s Trace = 0.928; *F*(9, 393) = 28.83, *p* < 0.001, η*_*p*_*^2^ = 0.309]. Compared with all career groups, the combined artistic professions presented significantly higher percentage of individuals marking intrinsic motives [*F*(3, 131) = 51.76, *p* < 0.001, η*_*p*_*^2^ = 0.542], lower percentage of individuals marking extrinsic motives [*F*(3, 131) = 129.74, *p* < 0.001, η*_*p*_*^2^ = 0.748], and higher intrinsic to extrinsic ratio [*F*(3, 131) = 84.27, *p* < 0.001, η*_*p*_*^2^ = 0.659] (for details, see Figure 5).

The *post-hoc* test with Bonferroni correction showed differences between Artistic careers and all other career groups in intrinsic motives (all *p*’s < 0.001), in extrinsic motives (all *p*’s < 0.001), and in the intrinsic to the extrinsic ratio (all *p*’s < 0.001). Similarly, a higher percentage of individuals within non-humanities careers reported intrinsic motives for career choice than All careers group (*p* = 0.019), and Humanities non-artistic careers (*p* < 0.001). The Non-humanities careers group also presented a lower percentage of individuals reporting extrinsic motives than the All-careers group, and Humanities non-artistic (all *p*’s < 0.001). There was no other difference among the comparison groups. Among all applicants, the intrinsic to extrinsic ratio showed that intrinsic motives (“personal aptitude” and “personal fulfillment”) were 2.60 times higher than extrinsic factors (“social prestige,” “social contribution,” “lower concurrence,” “attractive job market,” and “attractive salary”). Among Non-humanities careers the intrinsic to extrinsic ratio was 3.74, among Humanities non-artistic careers it was 2.24, but among applicants for artistic careers, intrinsic motives were 10.81 times higher, which suggests a specific overall motivational profile (see Figure 5).

In Figure 5, the cluster **“All careers”** includes 62 different careers; **“non-humanities”** include Chemistry, Dentistry, and Physical Education; **“Humanities Non-artistic”** include History, Philosophy, Pedagogy, and Social Sciences; **“Artistic”** include Music, Dance, Scenic Arts, Artistic Education/Visual Arts, and Literary Studies.

**FIGURE 6:**
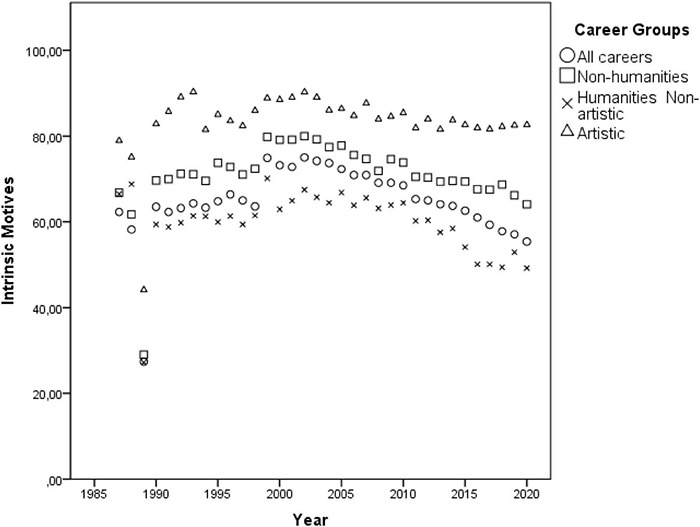
Dispersion of the averaged percentages of individuals indicating *intrinsic* motives of career choice by the cluster of careers throughout the years 1987 and 2020.

**FIGURE 7:**
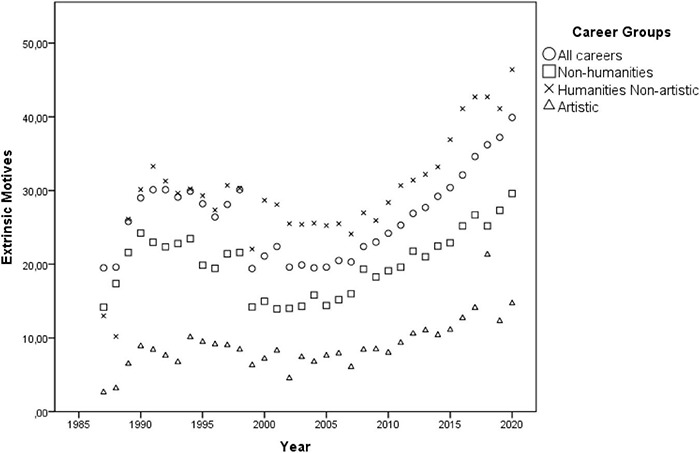
Dispersion of the averaged percentages of individuals indicating *extrinsic* motives of career choice by the cluster of careers throughout the years 1987 and 2020.

The multivariate GLM model indicated no effect of the year on intrinsic motives [*F*(1, 131) = 0.357, *p* = 0.551, η*_*p*_*^2^ = 0.003], but an effect on extrinsic motives [*F*(1, 131) = 48.34, *p* < 0.001, η*_*p*_*^2^ = 0.270], and on the intrinsic to extrinsic ratio [*F*(1, 131) = 17.43, *p* < 0.001, η*_*p*_*^2^ = 0.117] throughout the 34 consecutive application period [Pillai’s Trace = 0.370; *F*(3, 129) = 25.29, *p* < 0.001, η*_*p*_*^2^ = 0.370] (see Figures 6, 7). The Pearson correlation confirmed that the years did not correlate with intrinsic motivations [*r*(136) = 0.35, *p* = 0.683], but was positively associated with the extrinsic ones [*r*(136) = 0.292, *p* < 0.001] and negatively with the intrinsic to extrinsic ratio [*r*(136) = –0.208, *p* = 0.015], suggesting the overall temporal stability of intrinsic motives (see Figures 6, 7).

**FIGURE 8:**
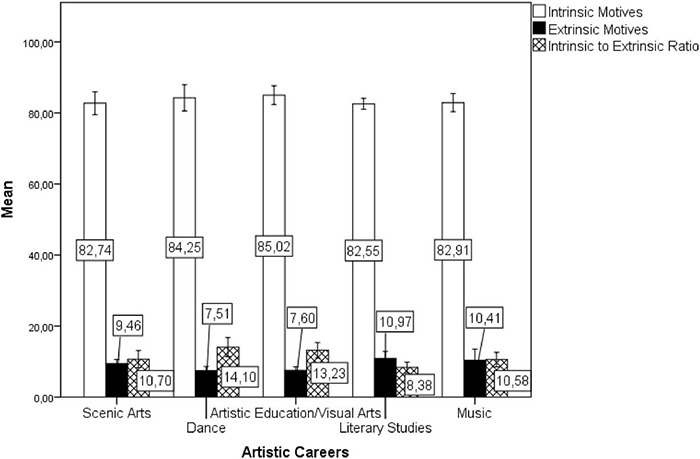
Averaged percentages of individuals indicating intrinsic or extrinsic motives of career choice by Artistic careers in the long-standing question (1987–2020). Error bars are ± 2 SEs.

Finally, the author compared the motives among the artistic careers to test for the uniformity of pattern within the artistic career group. The author also performed a multivariate GLM with the two classes of motives (intrinsic × extrinsic) and the intrinsic to extrinsic ratio as dependent variables, the artistic careers as independent factors (Music, Dance, Scenic Arts, Artistic Education/Visual Arts, and Literary Studies), and the year (34 consecutive application periods) as a covariate. The model showed no difference [Pillai’s Trace = 0.101; *F*(12, 432) = 1.26, *p* = 0.241, η*_*p*_*^2^ = 0.034] among the artistic courses in the intrinsic motives [*F*(4, 144) = 0.659 *p* = 0.621, η*_*p*_*^2^ = 0.018] extrinsic motives [*F*(4, 144) = 2.574, *p* = 0.040, η*_*p*_*^2^ = 0.067] or the intrinsic to extrinsic ratio [*F*(4, 144) = 2.333, *p* = 0.046, η*_*p*_*^2^ = 0.065]. Although the subsequent univariate tests indicated a small marginally significant difference among artistic careers in extrinsic motives and the intrinsic to extrinsic ratio the Bonferroni *post hoc* showed no differences among them (lowest *p* = 0.091) (for details, see Figure 8).

The same multivariate GLM model showed no effect of the year (34 consecutive application periods) [Pillai’s Trace = 0.371; *F*(3, 142) = 27.924, *p* < 0.001, η*_*p*_*^2^ = 0.371] on intrinsic motives [*F*(1, 144) = 2.704, *p* = 0.102, η*_*p*_*^2^ = 0.018], but an effect on extrinsic motives [*F*(1, 144) = 46.423, *p* < 0.001, η*_*p*_*^2^ = 0.244] and on the intrinsic to extrinsic ratio [*F*(1, 144) = 46.482, *p* < 0.001, η*_*p*_*^2^ = 0.244]. The Pearson correlation confirmed that the years did not interact with intrinsic motivations [*r*(150) = 0.177, *p* = 0.155], but only positively with the extrinsic ones [*r*(150) = 0.495, *p* < 0.001] and negatively with the intrinsic to extrinsic ratio [*r*(150) = -0.504, *p* < 0.001], suggesting the temporal stability of the intrinsic motives. Interestingly, among artistic careers there was no correlation between intrinsic and extrinsic factors [*r*(150) = -0.131, *p* = 0.109] (see Figures 6, 7).

## Discussion

The author tested whether artistic motivation as reflected from the reasons to apply for university artistic courses is intrinsic, domain-specific, and temporally stable in two studies using each different question of a large decades-long real-life public Brazilian database from university entrance applications. Using an unobtrusive method, the author analyzed the primary reasons to apply for a career according to a question involving 403,832 late-adolescents between 1987 and 1998 from 44 different careers and 18 different Brazilian cities (Study I); and according to a question involving 1,703,916 late-adolescents between 1987 and 2020 from 62 different careers and 35 different Brazilian cities (Study II). It comprised the artistic areas of Music, Dance, Drama, Visual Arts, and Literary Studies. General results from both studies supported a domain-specific, temporarily stable, and intrinsically sourced artistic motivation, consistent with its possible evolved nature. Across both studies, individuals applying for the combined artistic professions presented a significantly higher percentage of intrinsic factors (e.g., personal taste/aptitude/fulfillment) and a lower percentage of extrinsic factors (e.g., the influence of media/teacher/family, ample possibilities of salary, social prestige/contribution) than other careers groups. Among the total of applicants, the intrinsic to extrinsic ratio showed that intrinsic motives were 2.60–6.35 times higher than extrinsic factors, but among artistic applicants they were 10.81–28.38 times higher, suggesting a specific overall motivational profile. There was no difference in intrinsic motivation among different artistic careers suggesting a single-core global artistic motivational profile. Overall, the period interacted only with extrinsic motivations in only the longstanding question, suggesting that intrinsic factors are more temporally stable than extrinsic ones.

Both studies supported the hypothesis that artistic motivation is rather intrinsic, by showing that the combined artistic professions presented significantly higher intrinsic and lower extrinsic motivations than other groups of careers. These results corroborate studies showing that intrinsic reasons, such as personal interest in the artistic material itself, desire to express themselves artistically (McCarthy and Jinnett, 2001), recreation and aesthetics (Swanson et al., 2008), and self-expression and aesthetic experience (Chong, 2010) are among the primary reasons individuals report when engaging in artistic activities. This is in line with other evidence showing intrinsic artistic motivation, such as the intrinsically spontaneous, compulsive, and higher sustained artistic production in some patients with brain-damage (Zaidel, 2014; Midorikawa and Kawamura, 2015; Abraham, 2019). Further, experimental studies show that unrewarded motivation leads to better aesthetic value, while extrinsic motivation is somewhat detrimental (Amabile, 1982; Amabile and Gitomer, 1984; Amabile et al., 1986). Moreover, aesthetics and artistic activities are among the most popular choices of the spontaneously pursued free-time activities (Chalip et al., 1996; Walker and Scott-Melnyk, 2001; Chamorro-Premuzic and Furnham, 2004; Furnham and Chamorro-Premuzic, 2004; McManus and Furnham, 2006; Chong, 2010). The convergence of results from various lines of investigation makes a strong case in support of the rather intrinsic nature of artistic motivation as predicted from an evolutionary perspective (Morris, 1962; Dutton, 2009; Hogh-Olesen, 2018).

It is noteworthy that the effect size was bigger for the low extrinsic artistic motivation than for the high intrinsic artistic motivation. Thus, together with the increased intrinsic motivation, there is a major lack of external rewards for deciding to be a professional artist, which contradicts the “hunger for status” non-adaptive hypothesis (Pinker, 2004). Proximate sociocultural factors might also play a role in contributing to the obtained pattern of results. In Brazil and much of North America, art courses are elective as extra-class activities in high schools, hence favoring intrinsic interests and lacking the mandatory aspect of the core disciplines. In fact, in order to be approved in the test of specific artistic abilities, applicants needed to take elective preparatory courses. Further, being labeled talented or gifted by others might offer better opportunities, teachers, and curriculum (Berlin, 2009). This positive social context might reinforce the students’ perception of innate artistic talent (Evans et al., 2000), which can further positively influence their high intrinsic motivation. There is also a pervasive notion that arts are not a lucrative or sensible career path (Abbing, 2008), so much so that music students report that family and friends actively discourage their musical development (Evans et al., 2000), which can contribute to the obtained result of low extrinsic artistic motivation. Although relevant, the sociocultural factors likely do not fully explain the pattern of present results. That is because in some instances there was no correlation between intrinsic and extrinsic measures, and there are indeed important biological components to artistic talent including strong intrinsic motivation, referred to as “rage to master” (Winner and Drake, 2018), including multigenerational continuity (Perrone et al., 2010), and medium to high heritability in the choice of creative professions (Roeling et al., 2017). A twin-based study even showed that as musical ability, a musical practice also is substantially heritable (40–70%), and the association between both practice and ability was predominantly genetic so that identical twins differing in the amount of practice did not differ in their ability (Mosing et al., 2014). Therefore, it is most likely that both biological and cultural factors converge and complement each other (cf. Varella et al., 2012) affecting both intrinsic and extrinsic motivational aspects generating to the obtained overall pattern of results, as captured by the artistic specificity of the intrinsic and extrinsic ratio.

Both studies supported the hypothesis that artistic motivation is domain-specific by showing that the intrinsically sourced motivational profile of individuals applying for artistic careers is uniquely increased and distinct from other non-artistic careers: humanities, non-humanities, and also the total of careers. Across the two studies and investigated questions, the relative proportion between intrinsic and extrinsic reasons is around 4–4.5 times higher among applicants for artistic careers than for the total of applicants. Furthermore, the intrinsically sourced motivational profile did not differ among different artistic careers, which indicates that the same specific artistic motivation is globally underlying different artistic modalities. These findings support various taxonomies and classificatory schemes that consider artistic/aesthetic as a specific and legitimate domain within the field of vocation interests (Kuder, 1948; Guilford et al., 1954; Moloney et al., 1991; Holland, 1997; Su et al., 2019), of leisure practices (Dumazedier, 1988; Scott and Willits, 1998; Mingo and Montecolle, 2014), and even of engagement with beauty (Diessner et al., 2008). The convergent evidence pointing to domain-specificity is a hallmark of mental adaptation (Cosmides and Tooby, 1994; Andrews et al., 2002; Barrett, 2008; Tooby and Cosmides, 2015) that should be taken into account when analyzing the evolved nature of artistic propensities (cf. Varella et al., 2010; Valentova et al., 2019, focusing on musicality).

The hypothesized temporally stability also received support from both questions. The time period of 8 and 33 years, respectively, did not interact with intrinsic motives from both questions and with extrinsic motives from one question, suggesting overall temporal stability, particularly of the intrinsic motivation, at least within the three decades’ time scale. The historical stability is aligned with studies showing lifetime individual stability of interests. Ranging from the highly sustained artistic expression of some patients with brain damage (Abraham, 2019), passing through the stability of vocation interests (Lubinski et al., 1995; Waller et al., 1995), stability of leisure interests (Waller et al., 1995), and stability of engagement with artistic beauty (Diessner et al., 2008) throughout periods of individuals’ life. Similarly, particular interests reflecting hands-on physical activities and self-expressive/artistic activities tend to be even more stable than scientific, social, enterprising, and clerical interests (Low et al., 2005). Moreover, the earliest human artistic manifestations date back to the Middle (d’Errico et al., 2009; Henshilwood et al., 2018) and Lower Paleolithic (Bednarik, 2014), which gives enough time for artisticality to get specialized and well stabilized (cf. Starratt and Shackelford, 2010).

The overall pattern of results did not support the non-adaptive hypothesis based on general curiosity, or “hunger for status,” as put forward by Pinker (2004). Contrary to the by-product predictions there were differences between artistic and non-artistic careers, intrinsic factors were more relevant than extrinsic ones, and the period mostly did not influence artistic motivation. This fails to corroborate the non-adaptive hypothesis does not mean that curiosity and status-seeking are not related to the artistic domain, only that there might be a more basic and specific underlying motivational factor at play. The confluence of present results from both studies together with related previous evidence in corroborating the hypotheses that artistic motivation is rather intrinsic, domain-specific, and at least three decades historically stable offers solid empirical support for the notion that artistic motivation is an evolved aspect of human nature (cf. Morris, 1962; Dutton, 2009; Hogh-Olesen, 2018).

Moreover, the present study found that the same intrinsic motivational factors influence the five artistic modalities (viz. Scenic Arts, Visual Arts, Literary Studies, Music, and Dance). This offers further evidence to consider art as a legitimate global and coherent dimension in which the different modalities evolved together served by the same motivational mechanisms (Dutton, 2009). Furthermore, analyzing a huge sample size, real-life data, unobtrusive method, and a considerable temporal span, the present study for the first time establishes that the average general vocational interest is around twice as often motivated by intrinsic than extrinsic reasons, though there is a general recent historical trend decreasing this ratio.

There are some limitations in the present study. The entrance exam system (i.e., COMVEST) did not maintain perfect consistency in the questions phrasing, the order, or the number of answer options. However, the official changes were minor and performed only once throughout the period, which was accounted for in the analyses. Further, the entrance exams system rounds the percentages of the data up to only one decimal place, so the author expects a .1 error rate in the given percentages. Also, the system does not show frequency results when there are less than five candidates in a given career of a given year. Hence, in some years, some music instrument modalities, such as clarinet or trombone, could not be added to the other modalities to create the overall Music career. Importantly, although the data were taken from a Latin American country, a non-Western English-speaking area, the data do not represent the general Brazilian (or world) population given its circumscribed location (7 Brazilian states out of 26, but mostly São Paulo state). The studies were also age-limited mostly to late-adolescents applying for full-time diurnal, but not nocturnal courses. Future studies should try to account for and expand beyond these limitations to replicate and further explore the design features of human artistic motivation in real-life scenarios.

## Conclusion

I aimed to celebrate the 150 years of Darwin’s legacy from “The Descent of Man, and Selection in Relation to Sex” (1871) by advancing the evolutionary knowledge regarding the nature, scope, and structure of artistic motivation. The author has analyzed massive sample sizes of real-life data, a three decades’ time span, and a diversity of artistic and non-artistic careers. The convergent results across both studies indicated a distinctive and highly intrinsically based artistic motivation, that is specific to all arts and temporally stable within the three decades’ time span. This is supported in the literature by various lines of investigations.

Although less prominent, extrinsic factors also play a role, particularly the decreased reliance on extrinsic factors of individuals applying to artistic careers. Socio-historical factors including the structure of the educational system which leaves artistic courses as elective, and the difficult career prospects in the job market may also contribute to the obtained pattern of results. Thus, both biological and sociocultural factors converge and complement each other influencing both intrinsic and extrinsic motivational aspects akin to what Darwin has put forward in 1871.

Therefore, the author concludes that artistic motivation presents at least three predicted features consistent with its possible evolved nature; it is highly intrinsically sourced, has domain-specificity, and has some temporal stability. Future studies should explore these features using different indicators of motivation and use different methodological approaches to probe for these and other possible aspects.

## Data Availability Statement

The data source for this research is *per se* open, so anyone can access http://www.comvest.unicamp.br/estatisticas-comvest/vestibulares/vestibulares-anteriores/ to choose which year one wants to access. The specific data used for this research were gathered from the socioeconomic questionnaire (e.g., for the year 2019 is http://www.comvest.unicamp.br/estatisticas/2019/quest/quest1.php) within COMVEST webpage at the session about statistics. One only needs to select the course and which question to access each part of the data per year. Further inquiries can be directed to the corresponding author.

## Author Contributions

MV conceived and designed the studies, searched the literature, gathered the data, organized and analyzed the data, and wrote the manuscript.

## Conflict of Interest

The author declares that the research was conducted in the absence of any commercial or financial relationships that could be construed as a potential conflict of interest.

## Publisher’s Note

All claims expressed in this article are solely those of the authors and do not necessarily represent those of their affiliated organizations, or those of the publisher, the editors and the reviewers. Any product that may be evaluated in this article, or claim that may be made by its manufacturer, is not guaranteed or endorsed by the publisher.

## Footnotes

1
www.comvest.unicamp.br

2
http://www.comvest.unicamp.br/estatisticas/1998/quest/quest1.php
